# Clinical predictors of inflammatory bowel disease in a genetically well-defined Caucasian population

**DOI:** 10.1186/1477-5751-11-7

**Published:** 2012-01-23

**Authors:** Ziad M Kanaan, Maurice R Eichenberger, Surriya Ahmad, Clayton Weller, Henry Roberts, Jianmin Pan, Shesh N Rai, Robert Petras, E Brooks Weller, Susan Galandiuk

**Affiliations:** 1 Department of Surgery, The Price Institute of Surgical Research and the Section of Colorectal Surgery, University of Louisville School of Medicine, 550 S. Jackson St, Louisville, KY 40292, USA; 2 The Department of Bioinformatics and Biostatistics, University of Louisville School of Medicine, 505 S. Hancock St., Louisville, KY 40202, USA; 3 AmeriPath Institute of Gastrointestinal Pathology and Digestive Disease, Oakwood village, OH 44146, USA

**Keywords:** Inflammatory bowel disease, Crohn's disease, Ulcerative Colitis, SNP, *NOD2*, *IL23r*, *OCTN1*, *IGR*

## Abstract

**Background:**

Crohn's disease (CD) and ulcerative colitis (UC), the two main types of inflammatory bowel disease (IBD), are multifactorial conditions of unknown etiology. The objective of this study is to examine the combined gene-environment interactions influencing IBD susceptibility in a well-defined Caucasian cohort in rural mid-America.

**Methods:**

Patients were diagnosed to have CD or UC using conventional radiologic, endoscopic, and/or histopathologic findings. Histological diagnosis was made by a single specialist gastrointestinal pathologist with a particular interest in IBD. Information regarding cigarette smoke exposure was obtained by administration of the Behavioral Risk Factor Surveillance System Survey (BRFSS) to all patients. Genomic DNA was extracted from peripheral blood leukocytes, and polymerase chain reaction (PCR) amplification and genotyping were performed for 11 Single Nucleotide Polymorphisms (SNP) in *NOD2*, *IL23r*, *OCTN1 *genes along with *IGR*.

**Results:**

Our cohort consists of 1196 patients: 435 controls, 485 CD patients, and 276 UC patients. Only patients with genotype data for at least 7 of 11 SNPs were included in our data analysis. The control groups for all 11 SNPs were in Hardy-Weinberg Equilibrium. In genotype-association SNP analysis, all *NOD2 *SNPs (rs5743293, rs2066844, rs2066845) and the *IL23r *SNP (rs11465804) showed a significant association to IBD (*p *< 0.03). A multiple gene-interaction analysis showed an association between *NOD2 *and *IL23r *with UC (*p *= 0.04). There were no associations between any *OCTN1 *and *IGR *SNPs and IBD in this cohort. A multivariable logistic regression analysis showed that female gender, "current" or "former" smoking status, family history of IBD, and *NOD2 *SNP minor alleles were associated with CD.

**Conclusion:**

IBD remains to be challenging to properly diagnose, characterize, and treat. Our study proposes a combined genetic, phenotypic, and environmental approach in an attempt to better understand IBD. Previously demonstrated associations between OCTN1 and IGR and IBD were not confirmed.

## Background

Inflammatory bowel disease (IBD) is a chronic inflammatory disorder of the gastrointestinal (GI) tract, comprised of Crohn's disease (CD) and ulcerative colitis (UC). Over the past century, Northern Europe and North America have witnessed a significant rise in incidences of IBD [1]. The inflammatory response in CD patients is described by transmural inflammation in any portion of the gastrointestinal tract while that of UC is usually limited to the mucosa and submucosa of the colon and rectum [2]. Although the exact pathogenesis is not completely known in IBD, our current understanding suggests a disease etiology dependent upon a multifaceted interaction between genetic, environmental, and clinical factors[3].

Evidence of genetic factors implicated in this condition is supported by increased rates of IBD in monozygotic twins, and the ethnic differences in IBD frequency [4]. In addition, different races and ethnic groups have different degrees of susceptibility to IBD [5]. A number of studies have shown an association between IBD susceptibility and the nucleotide-binding oligomerization domain 2 gene (*NOD2, also known as CARD15*), interleukin-23 receptor gene (*IL23r*), organic cation transporter novel type 1 gene (*OCTN1*), and the intergenic region (*IGR*) variants [6-9]. *NOD2 *gene mutations have been extensively studied and described in the literature in terms of IBD susceptibility. Loss-of-function mutations in *NOD2 *gene appear to be the most significant for development of IBD, although the mechanism that increases disease susceptibly is poorly understood. One report suggests that *NOD2 *contributes a protective function in host defense that when compromised elicits a loss in immune bacterial recognition [10]. The *NOD2 *gene on chromosome 16 has been specifically implicated in susceptibility to CD with 30-50% of Caucasian CD patients having variants of this gene [6]. The three *NOD2 *variant single nucleotide polymorphisms (SNPs) (rs2066844, rs2066845, and rs5743293) have been strongly associated with clinical presentation of inflammatory bowel disease [1,11].

Environmental factors are likely to contribute to IBD susceptibility; however, they are poorly understood and insufficiently described. The recent alarming rise in IBD incidence in the US points to changes in environmental factors rather than drastic alterations in allele frequency, since genetic remodeling could not occur over such a brief time [12]. It seems plausible that environmental influences play as significant a role as their genetic counterparts and their combined actions dramatically affect disease presentation [13].

The objective of this study is to examine the gene-environment interaction which may influence the causation of IBD. We aimed to:

1. Study the incidence of SNP variants in *NOD2, IL23r, OCTN1*, and the *IGR *genes in a unique well-characterized rural Caucasian IBD population.

2. Examine haplotype frequencies in *NOD2*, *IL23r*, and *IGR *genes in both CD and UC patients.

3. Explore the SNPs' interaction, their possible effect on IBD susceptibility, and report a possible signature interaction model that could differentiate CD and UC patients.

4. Report risk factors that could serve as clinical predictors of IBD susceptibility

## Results

### Population Description

For the purposes of our genetic and clinical comparisons, we only examined the Caucasian subset in order to maintain statistical and descriptive homogeneity. Our patient population was composed of 1196 unrelated patients (63% women): 435 controls, 485 CD patients, and 276 UC patients.

The mean age at time of IBD diagnosis was 31 years. Twenty-seven percent of patients reported a family history of IBD. The smoking status of the population was defined as follows; 21% as "current" smokers, 30% as "former" smokers and 49% as "never" smokers. Our CD patients were described using the Vienna classification system (Table 1). Sixty-five percent of UC patients had pan-colitis, 22% had left-sided colitis and 13% isolated proctosigmoiditis.

**Table 1:** Characteristics of CD patients using Vienna Classification

Characteristic	CD Patients*
***Vienna Age***	***N (%)***

A1 (< 40 yrs)	372 (77)

A2 (> 40 yrs)	111 (23)

***Vienna Behavior***	***N (%)***

B1 (Non-stricturing, non-penetrating)	110 (23)

B2 (Stricturing)	131 (27)

B3 (Penetrating)	240 (50)

***Vienna Location ***	***N (%)***

L1 (Terminal ileum)	129 (27)

L2 (Colon)	146 (31)

L3 (Ileocolon)	169 (35)

L4 (Upper GI)	32 (7)

*Based on available clinical data

### Analysis of Clinical Variables

Univariate analysis of UC *vs*. CD examined previously described relevant clinical factors associated with IBD. Gender, smoking status, number of IBD-associated operations, and family history of IBD were strong indicators of CD (*p <*0.002). Age at diagnosis above 27 years old was more predictive of UC (*p = *0.003).

### SNP and Haplotype Analysis

For SNP analysis, we included only data for patients successfully genotyped for at least 7 out of 11 SNPs. Thus, there were 909 patients remaining for analysis, as follows: 303 controls, 366 CD patients, and 223 UC patients. The control groups for all 11 SNPs were in HWE. Univariate analysis was performed on all 11 SNPs. In genotype-association SNP analysis, only the *NOD2 *SNPs (rs5743293, rs2066844, rs2066845) and *IL23r *SNP-rs11465804 showed a significant association with IBD (*p <*0.025) (Table 2). The minor alleles in *NOD2 *SNPs (rs5743293 and rs2066845) in addition to *IL23r *SNP-rs11465804, were associated with CD (*p ≤ *0.01), but not UC. The *NOD2 *SNP (rs2066844) minor allele was associated with both CD and UC (*p <*0.005), but was more significantly associated with CD. We also examined the genetic interaction between *NOD2 *and *IL23r *genes for UC and CD; this interaction was predictive for UC (*p = *0.04), but not for CD (*p = *0.63).

**Table 2:** Univariate Analysis of Allelic Association in SNPs

Single Nucleotide Polymorphism	Total # of Alleles (*Total *= 1644) (N, %)	Control (*Total *= 582) (N, %)	Crohn's Disease (*Total *= 654) (N, %)	Ulcerative Colitis (*Total *= 408) (N, %)	*p*-value
***NOD2 *- rs5743293**					***< 0.001^1^***

C	59 (4)	8 (1)	43 (7)	8 (2)	***< 0.0001^2^***

N	1499 (96)	546 (99)	561 (93)	392 (98)	***0.509^3^***

					***< 0.001^4^***

***NOD2 - *rs2066844**					***< 0.001^1^***

C	1377 (93)	466 (97)	545 (89)	366 (93)	***< 0.001^2^***

T	109 (7)	14 (3)	67 (11)	28 (7)	***0.004^3^***

					***0.042^4^***

***NOD2 *- rs2066845**					***< 0.001^1^***

G	1558 (98)	560 (99)	601 (96)	397 (99)	***< 0.001^2^***

C	38 (2)	6 (1)	27 (4)	5 (1)	***1.000^3^***

					***0.006^4^***

***IL-23r *- rs11465804**					***0.022^1^***

G	67 (4)	30 (5)	16 (3)	21 (5)	***0.010^2^***

T	1545 (96)	534 (95)	630 (97)	381 (95)	***0.948^3^***

					***0.019^4^***

*^1 ^*Global (Fisher's Exact Test)

*^2 ^*Control versus CD

*^3 ^*Control versus UC

*^4 ^*CD versus UC

The overall haplotype test was significant (*p <*0.0001) for *NOD2*, with significant differences in the frequencies of rare haplotypes, with both C-C-G (*p = *0.008) and C-C-C (*p = *0.018) being more associated with CD than with UC (Table 3). With IL23r gene, the C-A-C-G-G haplotype was associated with CD (*p = *0.009), while the T-A-C-G-G haplotype was associated with UC (*p = *0.023) (Table 4). *IGR *gene analysis showed there was no overall haplotype association with CD or UC (*p = *0.98 and 0.063).

**Table 3:** *NOD2 *Haplotype Association Analysis

	SNPs		Haplotype Frequencies				
				
		Haplotype	Control	CD	UC	CD and UC combined	*p-*value (CD vs. UC)
	rs574329 - rs2066844	C-C	1.34	6.46	1.97	4.75	***< 0.0001***

	rs574329 - rs2066845	C-G	1.47	5.3	1.97	4.02	***0.004***

***NOD-2 gene***		C-C	0	1.18	0	0.74	***0.027***

	rs2066844- rs2066845	C-C	1.13	3.54	1.09	2.64	***0.009***

	rs574329 - rs2066844- rs2066845	C-C-G	1.35	5.27	1.97	4.01	***0.008***

		C-C-C	0	1.19	0	0.75	***0.018***

**Table 4:** *IL23r *Haplotype Association Analysis

		Frequencies (%)				
			
SNPs	Haplotype	Control	CD	UC	CD and UC combined	*p-value* (CD vs. UC)
rs10489629 - rs11465804	G-G	3.91	2.25	4.52	3.16	***0.048***

rs1004819 - rs10489629 -rs11465804	C-G-G	3.94	2.06	4.36	2.95	***0.045***

rs1004819 - rs2201841 - rs11465804 - rs11209026	T-C-G-G	0.01	0.02	1.1	0.45	***0.014***

	C-C-G-G	0	0.353	0	0.002	***0.010***

rs1004819 - rs10489629 -rs11465804 - rs11209026	C-A-G-G	0.4	0.78	0	0.1	***0.016***

	T-A-G-G	0.03	0.006	1.34	0.61	***0.028***

rs1004819 - rs10489629 - rs2201841 - rs11465804	C-G-T-G	3.92	2.14	4.37	3.02	***0.049***

	C-A-C-T	4.97	4.07	7.19	5.1	***0.048***

rs1004819 - rs10489629 - rs2201841 - rs11465804 - rs11209026	T-A-C-G-G	0.01	0.02	1.12	0.41	***0.023***

	C-A-C-G-G	0	0.36	0	0.008	***0.009***

### Multivariate Analysis of Genetic and Clinical Predictors (CD vs. UC)

We used a multivariable logistic regression model comprising the most significant clinical predictors and the above mentioned SNP variants implicated in IBD (Table 5). The *IL23r *SNP-rs11465804 was not included in the regression analysis since its correlative affect in IBD was not as significant as its *NOD2 *counterparts. Female gender, current or former smoking status, IBD-associated surgery, family history of IBD, and frequency of all *NOD2 *SNP minor alleles were descriptive predictors of CD. Age at time of diagnosis ≥ 27 years was predictive of UC diagnosis.

**Table 5:** Multivariable Logistic Regression Analysis for UC *vs*. CD

Genetic and Clinical Variables		Ulcerative Colitis vs. Crohn's Disease		
		Estimate*	***p*-value**	**OR (95% CI)**

***Age at time of diagnosis***	≥ 27	0.32	***0.04***	1.38 (1.02-1.87)

	< 27	0		

***Gender***	Female	-0.38	***0.01***	0.68 (0.50-0.92)

	Male	0		

***Smoker Status***	Current	-1.63	***< 0.0001***	0.20 (0.13-0.30)

	Former	-0.81	***< 0.0001***	0.45 (0.32-0.63)

	Never	0	***< 0.0001***	

***Number of Operations***	Yes	-0.63	***0.002***	0.53 (0.36-0.79)

	No	0		

***Family History IBD***	Yes	-0.88	***< 0.0001***	0.42 (0.30-0.57)

	No	0		

***NOD2 - rs5743293 ***	C	-1.72	***0.0002***	0.18 (0.07-0.44)

	N	0		

***NOD2 - rs2066844***	T	-0.60	***0.03***	0.55 (0.33-0.93)

	C	0		

***NOD2 - rs2066845***	C	-1.30	***0.02***	0.271 (0.09-0.84)

	G	0		

* A positive estimate indicates the variable being more predictive of ulcerative colitis while a negative estimate is more predictive of Crohn's disease.

## Discussion

### I. Current Diagnosis of IBD

IBD is a complex genetic disorder based on a multifaceted interaction between specific genes and environmental factors. The definitive diagnosis and correct identification of IBD remains difficult. IBD diagnostic markers may be lacking in the earliest stages of disease [14]. Radiologic, endoscopic, and histological diagnostic approaches are commonly employed in identification of IBD; however, the inter-observer variability between pathologists calls into question their true clinical value. Distinction between CD and UC, particularly in the case of colonic IBD is important since surgical management varies in each disease, however, in many instances the pathological features overlap making differentiation problematic [15]. Particularly in CD where variability of histological presentation makes accurate diagnosis a challenging exercise, pathologists often differ on their individual assessments [16]. The apparent complexity of successful IBD diagnosis with current techniques demonstrates the immense value of developing a genetic and clinical screening tool.

### II. Clinical and Environmental Predictors of IBD

There are several established clinical descriptors associated with IBD. Our analysis identified age at diagnosis, gender, smoking status, and family history of IBD as being the most important clinical predictors to distinguish UC from CD. Previous reports indicate that these factors are significantly related to IBD, but failed to adequately describe their effects.

#### A. Age at Time of Diagnosis

Loftus *et al*. described a systematic review of North Americans with CD and UC in respect to patient age at time of diagnosis. The mean age for CD diagnosis ranged from 33 - 45 years [17] while UC patients were diagnosed 5 - 10 years [18,19] later. In concordance with these reports, the median age at time of diagnosis in our cohort was found to be 27 years. The age at time of diagnosis of ≥ 27 years age was found to be more predictive of UC patients (*p *= 0.04).

#### B. Gender

In a separate report, Loftus and Sandborn described the influence of gender on disease occurrence for CD and UC [20]. They reported an increased incidence of UC among men, and CD among women [20]. Our analysis concurs with this; the Caucasians in this study showed increased diagnosis of CD in women and UC in men (*p = *0.014). The female predominance in CD could possibly be attributed to unspecified hormonal interactions [1].

#### C. Family History of IBD

The positive correlation between family history and IBD incidence seems plausible; this is possibly due to a combination of underlying genetic and environmental factors. This was shown in a study done at a University Hospital in Finland where a comparison among IBD patients revealed 16% of CD patients and 14% of UC patients had at least one family member afflicted with IBD [21,22]. Our data strongly support these conclusions; i.e. a family history of IBD being more predictive of predisposition to CD than to UC (*p *< 0.0001).

#### D. Number of Surgeries

In addition to independent factors of gender, age, and family history, treatment approach may also be descriptive in IBD severity and progression. A North American population based study reported that 43% of patients with CD required major surgery involving incision, excision, and intestinal anastomosis compared to 40% for all UC hospitalizations [21]. Our results showed similar findings; CD patients were more likely to require surgery than their UC counterparts (*p = *0.015).

#### E. Smoking Status

Smoking was originally recognized as a risk factor for IBD susceptibility over 25 years ago [23,24]. A meta-analysis performed by Mahid *et al*. showed that 12 of 13 reports found that "current" smoking status could be protective against UC development. In contrast, "former" smoker status was found to be associated with an increased risk for the development of UC. A parallel analysis of Crohn's patients revealed 6 of 9 reports indicating an increased disease risk associated with having "ever smoked" [3]. Analysis of our patient cohort, "current" and "former" smoker statuses were both more associated with development of CD than UC (*p *< 0.001).

### III. Genetic Predictors of IBD

*NOD2 gene *is identified to be in the *IBD1 *locus on chromosome 16 as a CD susceptibility gene. Hugot *et al*. mapped the *IBD1 *linkage locus and identified *NOD2 *as the underlying IBD susceptibility gene in 2001[25]. Thirty non-conservative polymorphisms have been identified within this gene, but three single SNPs (rs2066844, rs2066845, and rs5743293) account for approximately 82% of the mutated alleles [26]. Replication studies have confirmed that these three SNPs are independently associated with disease [11,27,28].

The receptor for the proinflammatory cytokine, IL-23r, also appears to play a key role in the progression of the chronic inflammation found in CD by driving inflammation through its role in the T-helper 17 (Th17) response. Activated myeloid and T cells express the IL-23 receptor and the level of its expression may affect the way Th17 cells create excess mucosal inflammation [9].

#### A. NOD2 and IL23r SNPs

In our patient population, all *NOD2 *variants and one *IL23r *variant were associated with IBD. After multivariable logistic regression analysis, only the *NOD2 *SNPs (rs2066844, rs2066845, and rs5743293) remained highly significant predictors of disease (*p <*0.001), and in this context all were descriptive of CD, in agreement with previous reports. A meta-analysis conducted by Economou *et al*. found the same three *NOD2 *variants to be distinguishing risk factors for CD. According to their analyses, SNP rs5743293 carries a four-fold increase, rs2066845 a three-fold increase, and rs2066844 a two-fold increase of developing CD in non-Jewish descent Caucasians. They further reported that the combination of two of these *NOD2 *variants resulted in an increased odds of developing CD of 17 times the normal population [29]. In contrast to *NOD2 *SNPs, we did not find the same genetic importance associated with *IL23r *polymorphisms in terms of describing and characterizing IBD. Although only one *IL23r *SNP was marginally significant in our univariate analysis, another variation of this allele yielded notable results when present with a *NOD2 *variant. A combination of *NOD2 *rs2066844 and *IL23r *rs2201841 was descriptive of patients with UC compared to controls (*p = *0.04). No such association was found for CD patients.

#### B. Haplotype Analysis for NOD2 and IL23r

Another potential differentiator between CD and UC is haplotype analysis. Previous reports implicated numerous *NOD2 *and *IL23r *haplotype sequences associated to CD [30,31]. The exact mechanism for disease pathogenesis in terms of these *NOD2 *and *IL23r *SNPs remains unclear, but an understanding of how allelic combinations alter the course of disease may prove useful. In our results, we found two haplotype sequences in *NOD2 *associated with CD: C-C-C and C-C-G (rs5743293-rs2066844-rs2066845). With respect to the IL23r gene, one haplotype was associated with CD and one with UC: Haplotypes C-A-C-G-G with CD and T-A-C-G-G with UC (rs1004819-rs10489629-rs2201841-rs11465804-rs11209026). Although potentially useful as genetic descriptors of CD and UC, the rarity of these haplotypes makes their clinical use unlikely.

## Conclusion

Inflammatory bowel disease remains difficult to properly characterize, diagnose, and treat. Understanding the combined interactions between clinical, environmental, and genetic factors could serve as key in identifying IBD predictors as well as in properly differentiating between UC and CD. Our report implicates "female gender", "current" or "former" smoking status, positive family history of IBD, and *NOD2 *SNP minor alleles to be associated with CD. Clinically, this will help physicians identify patients with high risk for IBD development, differentiate CD from UC patients, and eventually help better tailor their medical/surgical treatment approach.

## Methods

### Population Selection and Classification

This study was approved by the University of Louisville Institutional Review Board (IRB). Written informed consent was obtained from all subjects. Genetic and clinical information was stored in a password-protected, prospectively maintained HIPAA compliant database. Patients were derived from a clinically well-described university-based IBD practice [4] and from a small relatively rural geographic area consisting of the state of Kentucky and southern Indiana. Our cohort is composed of 1337 patients (37% males and 63% females). It is comprised of unrelated individuals of various races (90% Caucasian, 7% African American, and 3% Asians). For the purpose of this study, we decided to study IBD in a uniform well-described group of patients, specifically Caucasians (n = 1196). These patients were not studied for IBD before. There is a profound environmental effect in our studied population as they are derived from one of the areas in the United States with the highest rates of adult cigarette smoking. Additionally, thirty seven percent of all IBD patients in our cohort have a positive family history of IBD, defined as a first or second degree relative with IBD, as compared to only 9% of controls. Perianal CD (PCD) occurred in 147 (46%) of CD patients. All patients were diagnosed to have IBD, using conventional radiologic, endoscopic, and/or histopathologic findings. In cases of IBD colitis, the diagnosis was confirmed by a single specialist gastrointestinal pathologist with a particular interest in IBD. CD patients and their clinical characteristics, in terms of age at diagnosis, disease location, and behavior were classified according to Vienna classification [32]. Disease location in UC was classified as rectosigmoid, left-sided, or Pancolitis. Comparator patients to function as controls included patients from the same geographic area seen for non-inflammatory, non-neoplastic disorders including hemorrhoidal disease and screening colonoscopy.

### Characterization of Environmental Risk

This included smoking status and it was verified by utilizing a validated Behavioral Risk Factor Surveillance Survey (BRFSS) that was prospectively administered. [24] "*Current smoking" *was defined as having smoked 100 cigarettes in one's lifetime and smoking every day or some days over the past six months.^24 ^"*Former smoking" *was defined as having smoked 100 cigarettes in one's lifetime but currently no longer smoking at all. [24] A "*non-smoker" *is defined as someone who has not smoked 100 cigarettes in his or her lifetime and who does not currently smoke.^24 ^The category "e*ver smoking" *includes individuals from both the current and former smoking groups, while those who "*never smoked*" includes only non-smokers. [24]

### Characterization of Clinical Data

Clinical data included gender, family history of IBD and cancer, age at time of diagnosis, presence of extra intestinal or perianal disease, number of IBD-associated surgeries (as a surrogate index for disease severity), and recurrence in CD.

### Characterization of NOD2, IL23R, OCTN, and IGR Polymorphisms

Peripheral blood was obtained and genomic DNA was then extracted with a Puregene^® ^DNA extraction kit (Gentra Systems Inc., Minneapolis, MN). Polymerase chain reaction (PCR) amplification and genotyping were performed on an ABI prism 7300 sequence detection system (Applied Biosystems^©^, Foster City, CA). TaqMan^® ^SNP-specific PCR primers and fluorogenic probes were obtained from by Applied Biosystems (Applied Biosystems^©^, Foster City, CA). The fluorogenic minor groove binder (MGB) TaqMan^® ^probes were labeled with a reporter dye, either FAM ([5-(&6)-carboxyfluorescein]) or VICs (a proprietary fluorescent dye produced by Applied Biosystems) specific for the wildtype and variant alleles of each of the following SNPs[33,34] (additional file 1):

1- **Three *NOD2 *SNPs**: rs5743293, rs2066844, rs2066845.

2- **Five *IL23r *SNPs: **rs1004819, rs10489629, rs2201841, rs11465804, and rs11209026.

3- **Two *IGR *SNPs**: rs2522057 and rs7705189.

4- **One *OCTN *SNP: **rs1050152.

### Statistical Methods

Descriptive statistics related to genetic and clinical characteristics were produced for the entire cohort. Categorical variables were compared using the Pearson Chi-square test (or Fisher's Exact test) for contingency tables [34]. The *t*-test or Wilcoxon rank sum test was used to test the cohort for continuous variables. We also fit the univariable and multivariable logistic regression models for the probabilities of patients in "CD" or "UC" groups about their possible predictors [35]. Furthermore, we examined haplotype associations between CD and UC.

We performed univariate analysis of our control population compared against CD and UC as well as CD against UC patients. Based on this analysis, we selected the following most significant IBD clinical predictors: age at time of diagnosis, gender, smoking status, IBD-associated surgery, and family history of IBD as descriptive of the IBD subtype. The age at time of diagnosis was dichotomized as < 27 or ≥ 27 years (as the median age at time of diagnosis was 27). Using these clinical criteria, we performed multivariable logistic regression analysis combining both significant genetic and clinical predictors.

We explored the genotype association, allelic association, and Hardy-Weinberg Equilibrium (HWE) test for 11 SNPs using the Pearson Chi-square test. For allelic association analysis of the 11 SNPs, we used the binary logistic regression method to estimate their odds ratios and 95% confidence intervals for patients with CD or UC as compared to control group. Odds ratios and 95% confidence intervals were estimated using binary logistic regression. Since all the studied genes had already been identified as associated genes for IBD, CD and UC susceptibility, no multiple testing corrections were required. Haplotype analysis was conducted for all the SNPs of *NOD2*, *IL23r *and *IGR*. All calculations were performed with Statistical Analysis Software (SAS) program [34,36]. A *p*-value less than or equal to 0.05 was set to be significant.

## Competing interests

The authors declare that they have no competing interests.

## Authors' contributions

SG, ZK, and MRE completed the study design. ZK, SA, and SG drafted the manuscript. SG revised the manuscript for intellectual content. CW and HR completed the genetic analysis. EBW completed the acquisition of the clinical data. JP and SNR completed statistical data analysis. RP completed the histopathological review for enrolled inflammatory bowel disease patients. In addition, all authors have seen and approved the final version of this manuscript for submission.

## Supplementary Material

Additional file 1 **The four studied genes (*NOD2, IL-23r, OCTN1*, and *IGR) *along with the corresponding SNPs**. A list of the studied Single Nucleotide Polymorphisms (SNPs) in each gene of interest along with their reference numbers. Click here for file
