# Supplementary material for: Clinical predictors of inflammatory bowel disease in a genetically well-defined Caucasian population
Source: J Negat Results Biomed. 2012 Jan 23;11:7. doi: 10.1186/1477-5751-11-7 (PMC3292469; doi:10.1186/1477-5751-11-7)
Supplement: Additional file 1 — The four studied genes (NOD2, IL-23r, OCTN1, and IGR) along with the corresponding SNPs. A list of the studied Single Nucleotide Polymorphisms (SNPs) in each gene of interest along with their reference numbers. [file 1477-5751-11-7-S1.DOC]

**Additional table 1**

| **Gene** | **Reference SNP (rs) Number** | **Alternative Names** |
| --- | --- | --- |
|  | rs5743293 | - 4377981insC - 3020insC |
| ***NOD2*** | rs2066844 | - 4360125C>T - 2104C>T - Arg702Trp |
|  | rs2066845 | - 4370739G>C - 2722G>C - Gly908Arg |
|  | rs1004819 | - 37642131G>A - 2380G>A |
|  | rs10489629 | - 37660267T>C - 955+2936T>C |
| ***IL23r*** | rs2201841 | - 37666120A>G - 956-8194A>G |
|  | rs11465804 | - 37674444T>G - 1045+41T>G |
|  | rs11209026 | - 37677876G>A - 1142G>A - Arg381Gln |
| ***OCTN1*** | rs2522057 | - 40115819G>C |
|  | rs7705189 | - 39937230A>G |
| ***IGR*** | rs1050152 | - 39990192C>T - 1507C>T - Leu503Phe |
